# Case of Encysted Tumor in the Abdomen

**Published:** 1823-12

**Authors:** George Gregory

**Affiliations:** Senior Physician of the St. George's and St. James's General Dispensary.


THE LONDON
### Medical and Physical Journal.

6 OF VOL. L.] DECEMBER, 1823. [No. 298.

For many fortunate discoveries in medicine, and for the detection of numerous errors, the world is indebted to the rapid circulation of Monthly Journals; and there never existed any work, to which the Faculty, in Europe and America, were under deeper obligations, than to the Medical and Physical Journal of London, now forming a long, but an invaluable, series.—RUSH.

### ORIGINAL COMMUNICATIONS,
### SELECT OBSERVATIONS, &c.

ART. I.—*Case of Encysted Tumor in the Abdomen.* By GEORGE GREGORY, M.D. Senior Physician of the St. George's and St. James's General Dispensary.

SARAH COURT, thirty-six years of age, a widow, of spare habit of body, became a patient at the St. George's and St. James's Dispensary, on the 3d October, 1823. On her admission, she complained of considerable pain throughout the abdomen, constant, attended with frequent discharges of wind, and much weakness of the legs and thighs. Pulse quick; tongue foul; alvine evacuations regular; the catamenia had been absent for three years. Her present complaints were described as of four months' standing, and as becoming gradually worse. During the whole of this period, she had been subject to paroxysms of severe pain in the abdomen, recurring every three weeks, lasting for a few days, and relieved, for the most part, by a free discharge of flatus. On examination, the lower part of the abdomen appeared to be occupied by a firm swelling, which, though it gave no sense of fluctuation, was imagined to arise from some enlargement of the ovarium. The patient distinctly stated, that she could not refer its origin to either side in particular. She ascribed her complaints to extreme fatigue in her occupation as a washerwoman. Some warm aperients, that were directed, procured her some relief.

On the 14th October, I was requested to visit her at her own house. I found her suffering under an attack of severe pain of the abdomen, which was enlarged, tense, and very tender on pressure. She passed flatus in great abundance, but without any relief. She complained, also, of considerable difficulty of breathing, aggravated by the recumbent posture. Her tongue was loaded; the pulse quite imperceptible at the wrist; her bowels obstinately constipated. Cathartic medicines were freely administered, which procured several evacuations, and with them some slight relief. On the 16th, the symptoms recurred with great violence. The only position in which she could remain with any tolerable comfort, was kneeling by the side of the bed. Leeches were now applied to the abdomen, and fomentations renewed. Glysters, containing oil of turpentine, were thrown up; and to the medicines which she had previously taken were added the tincture of hyoscyamus, with very full doses of opium. By these means partial relief was obtained. On the evening of the 20th, the pulse having become more distinct, twelve ounces of blood were taken from the arm. The blood appeared cupped and buffy; but this measure procured for her only an hour or two of ease, while it materially diminished her general strength.

Fluctuation had been indistinctly perceived for some time, and, on the morning of the 21st, finding that all the plans of treatment hitherto devised had failed in obtaining for her any permanent benefit, I decided upon trying the chance of relief which the operation of tapping presented. Mr. Jeffreys introduced the trocar below the umbilicus, and about five pints of a transparent, brownish-coloured fluid were slowly drawn off. It was evident, during the operation, that some firm tumor was continually preventing the escape of the water; and no doubt could be entertained that the trocar had only penetrated the peritoneum lining the abdominal parietes, and that we had evacuated only that sheet of water which lay between it and the tumor. The degree of relief afforded by the operation was transitory and trifling. The patient suffered greatly by flatus the succeeding night, and opium now afforded the only solace which her medical attendants could devise. She sank rapidly after this, and died late in the evening of the 29th October, after experiencing for more than a fortnight, and almost uninterruptedly, the most excruciating pain I had ever witnessed.

The body was opened the following day, sixteen hours after death, by Mr. Jeffreys, in presence of Dr. James Johnson, myself, and a number of gentlemen whose attention had been attracted by the singular violence and remarkable character of the symptoms.

On dividing the abdominal parietes, a large firm tumor, of a black colour, was brought into view, attached by slight, and probably recently effused, bands of coagulable lymph, to the peritoneum lining the abdominal muscles. These adhesions were less firm towards the posterior part, so that, after separating the tumor from the parietes of the abdomen, it readily *turned out*, as the yolk of an egg separates from the white. This tumor occupied nearly the whole of the abdominal cavity from the ensiform cartilage to the pelvis, and was every where of a very dark colour, almost black. When cut into, it was found to contain a large quantity of a thick, fetid, chocolate-coloured fluid. The walls of the cyst were of unequal thickness: in many parts they were not less than two inches thick. Its external surface was *every where smooth,* making allowance for a loose covering of coagulable lymph. The internal surface of the coats of the cyst was rough and irregular. Its texture was throughout soft, spongy, and *rotten;* and, by many gentlemen present, was compared to that of a putrid placenta. Its whole weight, including that of the contained fluid, may probably have somewhat exceeded twelve pounds.

The uterus and its appendages were now cut out, and carefully examined. Both ovaria were found in a perfectly healthy state; and the uterus itself was free from disease.

The tumor being removed, gave us an opportunity of seeing that the peritoneum covering the bowels, liver, and other viscera, was every where of the same dark colour as the surface of the cyst; and it was in all parts covered with the same shreds of coagulable lymph as were witnessed in the tumor. The intestines were loosely glued together, and their coats appeared preternaturally thickened. The liver internally was of an ash-colour, and unhealthy texture. The stomach was not more than usually distended.

The heart and lungs were free from all traces of disease.

The case which I have now detailed appears to suggest two very interesting subjects of inquiry. First, what was the *nature* of the tumor, and how was it nourished? second, what was the nature, and what the occasion, of that peculiar *blackening* of the peritoneum, and of the surface of the cyst, which attended the inflammation of these parts?

It will not have escaped the notice of the reader, that the external surface of the tumor was every where *smooth;* that no one point could be detected at which vessels ran into it, or at which it had formed any but recent, and easily torn, connections. This circumstance led to the notion that the cyst had been originally of the nature of an hydatid; the coats of which, in the course of time, had taken on a diseased action, and acquired that degree of density in which we observed them. This hint was thrown out by Dr. James Johnson, and it seems to me to offer a rational explanation of the appearances. How the cyst was nourished, I am wholly at a loss to understand.

2. The second question is at least equally difficult of solution. The blackened appearance of the peritoneum could not be mistaken for sphacelus, for the membrane was *firm,* and the tinge was too universally diffused to admit of such an explanation. It has been suggested that this peculiar appearance is associated in its general pathology with the production of those masses of black matter occasionally deposited in different structures,

(particularly in cellular membranes,) to which the French have given the name of Melanosis. In this instance, however, the blackening was confined to the peritoneum, and to those parts occupied by inflammation; and, upon the whole, from my own observation, of a similar appearance in other bodies, (particularly as I have witnessed it at St. George's Hospital,) I am disposed to associate it with some peculiar kind of chronic inflammation. Whether this blackening inflammation derives its peculiarity from the degree of accompanying pain and tension, —or whether its source be to be sought in some more obscure state of the part affected,—are points on which I have speculated, but on which I am unable to come to any legitimate conclusion.

*8, Upper John-street, Golden-square;*

*November 6th,* 1823.

